# Scurvy: Rediscovering a Forgotten Disease

**DOI:** 10.3390/diseases11020078

**Published:** 2023-05-26

**Authors:** Mustafa Gandhi, Omar Elfeky, Hamza Ertugrul, Harleen Kaur Chela, Ebubekir Daglilar

**Affiliations:** 1 Department of Internal Medicine, University of Missouri, Columbia, MO 65211, USA; 2 Department of Medicine, University of Florida, Leesburg, FL 32611, USA; 3 Division of Gastroenterology and Hepatology, Charleston Area Medical Center, West Virginia University, Charleston, WV 25304, USA; he19md@gmail.com (H.E.);

**Keywords:** scurvy, vitamin C deficiency, gastrointestinal bleeding, mucosal ooze, vitamin C supplementation

## Abstract

Scurvy is a nutritional deficiency caused by low vitamin C levels that has been described since ancient times. It leads to a varied presentation, affecting multiple organ systems due to its role in the biochemical reactions of connective tissue synthesis. Common manifestations include gingival bleeding, arthralgias, skin discoloration, impaired wound healing, perifollicular hemorrhage, and ecchymoses. Although there has been a dramatic reduction in the prevalence of scurvy in modern times owing to vitamin C supplementation and intake, sporadic cases still occur. In developed countries, it is mainly diagnosed in the elderly and malnourished individuals and is associated with alcoholism, low socio-economic status, and poor dietary habits. Scurvy has been an unusual cause of gastrointestinal (GI) bleeding among other GI manifestations. It can be adequately treated and prevented via vitamin C supplementation.

## 1. Introduction

First described in 1550 BC in Eber’s papyrus, an Egyptian medical scroll, after being reported amongst soldiers and sailors who had minimal access to fruits and vegetables, vitamin C deficiency, also known as scurvy, is an old but not forgotten disease [1]. Scurvy has a deep historical significance and has plagued human populations for centuries. Ancient Egyptian, Greek, and Roman literature all provided detailed descriptions of the clinical signs and symptoms of scurvy. Scurvy decimated the European and British explorers of the Renaissance. During the Great Potato Famine, the American Civil War, the expedition of the North Pole, and the California Gold Rush, scurvy was a significant source of sickness and mortality throughout most of Europe. One of the first to show that sailors who spent months at sea might prevent scurvy by eating a diet high in vegetables was Captain James Cook. In a book titled *Treatise of the Scurvy*, James Lind, a Scottish naval surgeon, detailed his observations and research on scurvy aboard ships and described the effective treatment of scurvy with citrus fruits. As awareness of the importance of fresh fruits and vegetables in the diet increased, there was a decline in the prevalence of scurvy in the 18th century, especially after the establishment of the link between scurvy and vitamin C. Between 1928 and 1931, Szent-Gyorgyi extracted hexuronic acid from various sources such as cabbage, oranges, paprika, and adrenal glands. This substance was later identified as vitamin C and was discovered to have preventive properties against scurvy [2].

Vitamin C, also known as L-ascorbic acid, is a water-soluble nutrient and an essential dietary component that is vulnerable to heat, ultraviolet radiation, and oxygen. Ascorbic acid is involved in various body functions such as the absorption of iron, wound healing, and the formation of collagen. Although there are variations in the prevalence estimates, in some studies, it has been estimated that 7 ± 0.9% of the population of the United States suffers from scurvy [3]. Some of the risk factors that predispose these patients to the disease are chronic alcohol use, dietary insufficiency, and obesity [4,5]. Vitamin C is the cofactor for prolyl hydroxylase, which functions to stabilize the collagen molecule, and lysyl hydroxylase, which provides structural strength by cross-linking to the molecule [6]. As there is no long-term vitamin storage mechanism in our bodies, a lack of vitamin C can lead to vitamin C deficiency in as little as 1 to 3 months [7].

Scurvy has a variable clinical presentation due to its role in various bodily functions. Early symptoms can include fatigue, aching pain, irritability, and a loss of appetite. As the deficiency progresses, classic signs may appear, such as swelling of the gums, petechiae, bruising, and abnormal hair growth. Vitamin C deficiency weakens collagen triple-helix structures and fragile capillaries, which can lead to complications such as diffuse mucosal gastrointestinal bleeding [8]. Although coagulation parameters are usually normal, undiagnosed scurvy can result in significant bleeding and hospital burden, especially in post-operative cases [9]. This review article provides an overview of the history, epidemiology, pathophysiology, clinical manifestations, diagnosis, and treatment of scurvy, as well as recent advances in our understanding of this fascinating and important disease while also focusing on the gastrointestinal manifestations of this disease.

## 2. Biochemistry and Metabolism

Ascorbic acid is the enolic form of alpha-keto lactone, which shares a similar structure to glucose. Vitamin C refers to a group of compounds that have similar biochemical activities to ascorbic acid. Most mammals can synthesize vitamin C using glucose except for primates, fruit bats, and guinea pigs, as they lack the crucial enzyme, L-gluconolactone oxidase, that is necessary for this process.

The body contains a total pool of 1500–2500 mg of vitamin C, and the daily turnover rate is around 45–60 mg, which accounts for approximately 3% of the total amount. The half-life of vitamin C is 10–20 days. The absorption of vitamin C takes place in the ileum via an active transport mechanism The absorption of ascorbic acid occurs in the distal small intestine and relies on an energy-dependent active transport mechanism that can become saturated when the oral intake exceeds 180 mg/day; however, when consumed in typical dietary amounts of up to 100 mg/day, almost all the ascorbic acid is absorbed [10]. However, as dietary intake increases, the absorption rate decreases, and high pharmacologic doses of over 1000 mg/day may result in an absorption rate of less than 50%.

Dehydroascorbic acid is the oxidized form of ascorbic acid metabolism and can passively penetrate cellular membranes [11]. This form of vitamin C is preferred by erythrocytes and leukocytes as it is more readily absorbed by these cells. The ability of dehydroascorbic acid to penetrate cellular membranes plays a crucial role in vitamin C transport and metabolism in the body.

## 3. Pathophysiology

Ascorbic acid is an essential dietary vitamin for primates. Important dietary sources for humans include fresh fruits and vegetables such as citrus fruits, tomatoes, broccoli, strawberries, cabbage, potatoes, bell peppers, cauliflower, and spinach. Breast milk is an adequate source of vitamin C for infants [12]. Ascorbic acid is a reversible reducing agent that acts as an essential electron donor in several biochemical reactions and enzyme activities. Some of the biological processes that it is involved in are as follows:

- Collagen synthesis: Proline and lysine residues in the collagen structure must be enzymatically hydroxylated to produce the collagen found in the skin, blood vessels, and soft tissues. Prolyl hydroxylase and lysyl hydroxylase are enzymes that catalyze reactions, generating hydroxyproline and hydroxylysine, respectively. This reaction uses ascorbic acid as an electron donor. The inability to finish this step of collagen synthesis has adverse effects on bone and fibroblast functions, tooth development, and wound healing [13]. Furthermore, a deficiency in ascorbic acid causes epigenetic DNA hypermethylation and prevents the transcription of certain collagen types.
- Neurotransmitter synthesis: Ascorbic acid is a necessary cofactor for the enzyme dopamine-beta-monooxygenase, which hydroxylates dopamine to produce norepinephrine [14].
- Nitric oxide synthesis: The production of nitric oxide, a powerful vasodilator, is stimulated by ascorbic acid.
- Fatty acid transport: Ascorbic acid is necessary as an electron donor for the synthesis of carnitine. Long-chain fatty acid transportation across the mitochondrial membrane is a carnitine-dependent process [15].

Considering the widespread involvement of ascorbic acid in the formation and maintenance of soft tissues, scurvy results in numerous manifestations involving the skin and its appendages, impaired wound healing, dental and gingival disease, brittle bones, and hemorrhage relating to the loss of blood vessel integrity.

## 4. Epidemiology

Scurvy was traditionally described in sailors in older times, but there have been sporadic cases reported in recent times from underdeveloped regions without adequate nutritional support in at-risk populations. Although there is a variation in its global prevalence, the estimated overall prevalence of vitamin C deficiency in the US is about 5.9%, according to the 2017–2018 National Health and Nutrition Examination Survey (NHANES), whose aim was to assess the mean vitamin C serum levels and the prevalence of vitamin C deficiency (defined as a mean serum level of less than 11.4 μmol/L) [16]. The survey study sample consisted of 6740 civilians aged six years and older who were not living in institutions. These individuals were selected from the National Health and Nutrition Examination Survey (NHANES) conducted in 2017–2018 and were representative of 274,157,096 people in the United States. The researchers used multivariable linear and logistic regression analyses to investigate the predictive effects of various factors. They also compared the serum levels of Vitamin C and the prevalence of vitamin C deficiency in this sample with data from NHANES 2005–2006 using Student’s *t*-tests.

They discovered that women had a higher mean vitamin C serum concentration, while current smokers and obese individuals had a lower level. There was a decline in mean serum vitamin C levels without any significant change in the prevalence of vitamin C deficiency since the previous NHANES 2005–2006. The global incidence of scurvy can vary based on the socio-economic status of a region, with underdeveloped areas such as north India having an incidence as high as 73.9% [17].

## 5. Risk Factors

Given that vitamin C is an essential dietary nutrient for humans, manifestations of its deficiency are mainly related to the inadequate consumption or improper absorption of this nutrient in the small gut. Since 90% of ascorbic acid in the diet is from fruits and vegetables, a lack of these foods commonly leads to a deficiency. Since vitamin C is heat-sensitive, the manner of cooking also plays a role in the bioavailability of this nutrient in food [17]. Based on this, the risk factors or high-risk groups for vitamin C deficiency include the following [6]:

- Individuals with poor dietary habits who consume food of poor nutritional value;
- Limited access to or the inability to afford fresh fruits and vegetables;
- Alcoholism;
- Infants exclusively fed cow’s milk;
- Individuals with gastrointestinal disorders such as inflammatory bowel disease;
- Smoking was demonstrated as a significant risk factor in the NHANES [16];
- Low socio-economic status;
- Elderly individuals on a “tea-and-toast” diet;
- Eating disorders and psychiatric illness;
- Long-term use of certain medications such as corticosteroids or proton pump inhibitors, which can alter the absorption and bioavailability of vitamin C in the diet;
- Abdominal surgeries, such as small bowel resection or bariatric surgery, which affect gut absorption;
- Obesity;
- Dialysis [18].

A poor intake of vitamin-C-rich foods is a more obvious cause of deficiency when compared to obesity and abdominal surgeries. When talking about obesity as a cause of vitamin deficiencies, a change in the diet in the past few decades has led to the increased consumption of junk foods and fast foods such as pizzas, burgers, fried foods, and carbonated beverages, with a reduced intake of fresh fruits and vegetables; this has resulted in vitamin deficiencies resurfacing [19].

This change in dietary habits could be attributable to hectic work schedules, convenience, the cost of food, a sedentary lifestyle, and a lack of social support [20]. Bariatric surgeries such as sleeve-gastrectomy or gastric bypass can lead to an alteration in the acidic environment of the gut, causing impaired absorption [21]. The underlying causes of vitamin C deficiency and scurvy in children include psychiatric eating disorders such as avoidant/restrictive food intake disorder and anorexia nervosa, food insecurity, and neglect [22]. Another risk group in which scurvy has been reported is children with autism spectrum disorder who have a diet lacking fruits and vegetables [23]. Scurvy can occur in patients with excess iron secondary to hematological conditions such as thalassemia or sickle cell disease or a prior bone marrow transplantation [24]. Ferric deposition can accelerate the breakdown of ascorbic acid in the body; thus, iron overload can precipitate the manifestation of scurvy [25].

### Role of Vitamin C in the Immune System

Vitamin C plays an essential role in the regulation and function of the immune system. It affects the innate and adaptive immune system in a variety of ways.

- Barrier integrity: As previously discussed, vitamin C plays a major role in the synthesis of collagen, which is a component of soft tissue, including the epidermis and dermis. These skin layers actively accumulate ascorbic acid, suggesting that it plays a crucial role in maintaining the integrity of the skin and mucosal barriers to pathogens [26].
- Leukocyte Function: Studies have shown that neutrophils and lymphocytes accumulate ascorbic acid at concentrations 50 to 100 times higher than the plasma concentrations through active transport. The antioxidant properties of ascorbate within the cell are believed to protect the cells from free radicals from the oxidative burst. Additionally, vitamin C is also postulated to play a role in chemotaxis and neutrophil apoptosis [27].

## 6. Clinical Manifestations

The typical manifestations of scurvy begin to appear after 4 to 12 weeks of inadequate dietary ascorbic acid intake [14]. Non-specific symptoms such as fatigue, anorexia, and irritability may be seen when serum ascorbic acid concentrations dip below 20 μmol/L, but levels below 11.4 μmol/L indicate a substantial deficiency with which the more specific manifestations are observed [3].

Dermatological findings are generally specific for ascorbic acid deficiency and include follicular hyperkeratosis and perifollicular hemorrhage with petechiae and coiled hairs [28]. Ecchymoses, petechiae, and xerosis are other common skin findings. Initially, flat hemorrhagic skin lesions appear which may later coalesce and become palpable, especially on the lower extremities. These findings can be attributed to the reduced integrity of the dermal soft tissues due to impaired collagen synthesis, for which vitamin C is an essential component. Perifollicular hemorrhages usually occur in the lower extremities due to the capillaries’ vulnerability to hydrostatic pressure caused by gravity, which leads to “woody edema” [17]. The downregulation of tyrosinase enzyme activity from an ascorbic acid deficiency leads to an inhibition of melanin synthesis and skin discoloration in some patients [29]. Nail findings include koilonychia and splinter hemorrhages.

The musculoskeletal manifestations include arthralgias (typically of the knees, ankles, and wrists), muscle aches, hemarthrosis, and muscular hematomas [28,30]. By virtue of its role in biochemical reactions, vitamin C deficiency leads to alterations in structural collagen, deficient osteoid matrix formation, and increased bone resorption [30]. The musculoskeletal pain can be due to bleeding into the periosteum or muscles. Scurvy also causes the classic oral manifestations of gingivitis with bleeding and receding gums, as well as dental caries.

Fatigue, muscle weakness, malaise, arthralgias, loss of appetite, mood changes, peripheral neuropathy, and vasomotor instability are examples of generalized systemic symptoms that are commonly experienced with vitamin C deficiency. Dyspnea, hypotension, and sudden death have all been described as cardiorespiratory symptoms of scurvy, and it is hypothesized that these symptoms are brought on by a defective vasomotor response (especially given the role of ascorbic acid in nitric oxide synthesis) [30].

In the pediatric population, an acute limp can be the presenting musculoskeletal manifestation of scurvy owing to severe malnutrition [31]. A systematic review conducted by Trapani et al. on scurvy in the pediatric population revealed that 90% of children suffered from musculoskeletal complaints such as arthritis and lower limb pain, while about 33% had a limp and/or refused to walk [31,32]. Magnetic resonance imaging in children with scurvy can demonstrate certain characteristic features, such as a periosteal inflammatory reaction and local soft tissue swelling, in addition to sclerotic and lucent metaphyseal bands [24].

### Vitamin C and Lung Function

Ascorbic acid plays an important role in regulating the functioning of the pulmonary system. As an antioxidant, ascorbic acid plays an important role in the protection of lung tissue from reactive oxygen species. Akin to other leukocytes elsewhere in the body, the alveolar macrophages and alveolar type 2 cells concentrate vitamin C and scavenge reactive oxygen species to ameliorate oxidative damage [33].

Studies have shown promising data on the effect of high-dose intravenous ascorbic acid (HDIAA) in improving pulmonary function in those with severe COVID-19 pneumonia. The SARS-CoV infection is associated with a severe inflammatory response and a cytokine storm. Ascorbic acid helps maintain the integrity of the epithelial barrier and mitigates oxidative stress through its antioxidant properties [34].

## 7. Gastrointestinal Manifestations

The gastrointestinal (GI) tract is supplied by three major unpaired vessels that branch from the abdominal aorta, the celiac trunk, the superior mesenteric artery, and the inferior mesenteric artery. Branches from these major vessels then form anastomotic systems which, in turn, supply the gastrointestinal system and adjoining organs. Due to its high vascularity and large surface area, the GI tract is commonly investigated for bleeding in patients with anemia.

Recent studies have linked vitamin C to vascular function. In a study using cultured epithelial cells, d’Uscio et. al demonstrated the beneficial effect of vitamin C on vascular endothelial function [35]. This effect was mediated in part by the protection of tetrahydrobiopterin and the restoration of endothelial nitric oxide synthase enzymatic activity. There are a few hypothesized mechanisms through which vitamin C modulates vasorelaxation and increases nitric oxide synthesis or bioavailability. Firstly, vitamin C appears to recycle tetrahydrobiopterin, which is a co-factor for endothelial nitric oxide synthase. Endothelial nitric oxide synthase generates nitric oxide, which diffuses into the smooth muscle layer of the vascular wall and interacts with guanylyl cyclase and mediates vasodilation [36]. Secondly, vitamin C appears to regulate the activity of nicotinamide adenine dinucleotide phosphate (NADPH) oxidases and modulates the inflammatory response [37].

There have been infrequent cases reporting scurvy presenting as an overt gastrointestinal bleed. The literature on the gastrointestinal manifestations of scurvy is limited. (Table 1). Ohta et al. described a middle-aged man who had two years of anorexia and a diet deficient in fruits and vegetables when he developed hematochezia [37]. Erythema and intramucosal hemorrhage were discovered in the antrum and duodenum during an upper endoscopic evaluation. Similar observations of several intramucosal hemorrhages and redness in the rectum were found during a colonoscopy; these were biopsied for additional analysis. The rectal erythema was histologically examined, and the results showed inflammatory cell infiltration and fibrin exudation. Scurvy was determined to be the cause after additional testing of vitamin C levels, and hemorrhage was controlled after administration of a high dose of vitamin C. Callus et al. described a case of a 61-year-old man with a history of heavy alcohol use and limited food intake resulting in malnutrition [38]. He presented with upper gastrointestinal bleeding and had multiple bruises, poor dentition with bleeding gums, and telangiectasia upon examination. Blood tests showed low levels of vitamin C (0.21 mg/dL). Another case reported by Antunes et al. described a 40-year-old with a history of alcoholism and an unbalanced diet who presented with symptoms of polyarthralgia, bleeding gums, and episodes of hematochezia [39]. A physical examination revealed severe periodontitis with gingival hypertrophy and purplish areas consistent with necrosis. Blood tests revealed anemia and a vitamin C level of 0.14 mg/dL. A colonoscopy showed multiple intramucosal hemorrhages in the cecum and ascending colon. The patient was diagnosed with scurvy and treated with oral vitamin supplementation and adequate nutrition, resulting in complete clinical recovery within two months. Ertugrul et al. reported a case of refractory upper gastrointestinal bleeding in a morbidly obese patient mimicking portal gastropathy bleeding [40].

## 8. Diagnosis

The diagnosis of scurvy can be challenging as its symptoms may mimic those of other conditions. Additionally, individuals with scurvy may not present with all the classic symptoms. A combination of physical examination, medical history, dietary history, and laboratory tests is typically used to diagnose scurvy. During a physical examination, careful attention must be paid to signs of scurvy, such as swollen or bleeding gums, skin discoloration or bruising, and delayed wound healing. A medical history may be taken to determine risk factors for scurvy, such as dietary habits, chronic illness, and lifestyle factors. Laboratory tests can help confirm a diagnosis of scurvy. A blood test can measure vitamin C levels, which are typically low in individuals with scurvy. Symptoms of scurvy occur after the plasma concentration of ascorbic acid falls below 0.2 mg/dL; this value is usually calculated from plasma and leucocyte vitamin C levels [39]. Determining functional vitamin C status is challenging because there are no dependable indicators. Nevertheless, plasma and leukocyte vitamin C levels are commonly used to evaluate the status and are moderately associated with vitamin C consumption. Patients with a vitamin C deficiency commonly exhibit anemia, which may present as either iron-deficiency anemia (microcytic hypochromic) or a normochromic normocytic pattern [28]. In many cases, anemia in vitamin-C-deficient patients can be attributed to acute blood loss caused by defects in collagen synthesis. Such blood loss may occur in various soft tissue sites, including the gastrointestinal tract, joints, and muscles. Additionally, intravascular hemolysis has been observed in some cases, likely due to a decreased lifespan of red blood cells [28]. Overall, anemia is a frequent laboratory finding in patients with vitamin C deficiency and can have various underlying causes. Vitamin C plays a vital role in the absorption and metabolism of several nutrients that impact the production of red blood cells. One of the critical functions of vitamin C is aiding in the conversion of iron from the ferric form to the ferrous form, which is essential for the absorption of iron from the gastrointestinal tract. Moreover, scurvy may be associated with folate deficiency, and vitamin C helps to enhance the effect of folate in the production of red blood cells. Foods that are rich in vitamin C also tend to be high in folic acid, highlighting the importance of a balanced diet in preventing deficiencies in these essential nutrients [41].

Vitamin C deficiency is not commonly encountered in modern medicine. Therefore, a diagnosis of nutritional insufficiency accounting for a severe gastrointestinal bleed can only be considered with high clinical suspicion. Upper gastrointestinal bleeding has a wide range of differential diagnoses that may present similarly to vitamin C deficiency; therefore, it can be easily overlooked. These diagnoses include ulcerative gingivitis, blood dyscrasias, vasculitis and portal hypertensive gastropathy. Therefore, it is crucial to be aware of how uncommon causes of gastrointestinal bleeding, such as scurvy, manifest. The symptomatology can range from minor, non-specific signs to overt bleeding, including ecchymosis, bleeding gums, and a more serious hemorrhage.

In a study by Blee et al., it was found that patients in the hospital or undergoing surgery may have borderline levels of vitamin C which can further decrease due to a lack of oral intake post-surgery or other critical illnesses such as pancreatitis, sepsis, or multiple organ failure [10]. The study was conducted over a 12-month period in a surgical unit to identify patients with bleeding disorders. Out of the 12 patients who experienced widespread bleeding, none had a surgical cause; however, all had normal coagulation parameters but were found to have vitamin C levels below 0.6 mg/dL (the normal range is 0.6–2.0 mg/dL). Most of these patients had undergone abdominal surgeries, but significant bleeding was also observed in the cardiovascular and neurosurgical patients. The patients required a range of 2–13 units of blood transfusions, with 4.8 units being the average. It was also noted that 7 out of 12 of the patients who experienced widespread bleeding had poor oral nutrition prior to surgery.

## 9. Treatment

The treatment for scurvy is vitamin C supplementation and the reversal of the conditions that led to the deficiency. A wide range of replacement doses have been used successfully. For children, recommended doses are 100 mg of ascorbic acid given three times daily (orally, intramuscularly, or intravenously) for one week, then once daily for several weeks until the patient is fully recovered. Adults are usually treated with 300 to 1000 mg/day for one month [42].

The difficulty in treating hemorrhage caused by scurvy is not in treating the bleeding itself but rather in accurately diagnosing the condition. If scurvy is suspected, it can be effectively treated with high doses of Vitamin C. It has been reported that after just one replacement dose, gastrointestinal bleeding related to vitamin C deficiency will stop, and capillary stability will be established within 24 h. However, it can take up to 2–3 weeks for other symptoms of scurvy, such as skin lesions, to heal. The treatment of scurvy begins with high doses of Vitamin C: replacement is needed to replace the deficit in body stores. A recommended treatment course is an initial dosing of 1000 mg of intravenous ascorbic acid daily for 3 days, followed by further supplementation as needed with a dose of 250 to 500 mg twice daily for 1 month after discharge or longer if vitamin C cannot be to obtained via diet [40].

## 10. Prevention

Water-soluble vitamins such as vitamin C are stored in the body in very limited amounts and must be replenished through dietary intake. Ascorbic acid is most highly concentrated in certain body parts, including the pituitary gland, adrenal gland, brain, leukocytes, and the eyes. Unlike fat-soluble vitamins, which can be stored for long periods of time, water-soluble vitamins are quickly excreted from the body through urine. Therefore, it is important to ensure that an adequate amount of these nutrients is consumed on a regular basis to maintain healthy levels within the body. The United States RDA recommends the following daily intake amount of vitamin C [12]:

- Up to 6 months: 40 mg, as normally supplied through breastfeeding;
- From 7 to 12 months: 50 mg;
- From 1 to 3 years: 15 mg;
- From 4 to 8 years: 25 mg;
- From 9 to 13 years: 45 mg;
- From 14 to 18 years: 75 mg for males; 65 mg for females;
- From 19 years and older: 90 mg for males; 75 mg for females.

During pregnancy, it is recommended to consume 85 mg of vitamin C per day, increasing the amount to 120 mg during breastfeeding. Smokers require an additional 35 mg of vitamin C daily compared to non-smokers [12,43].

## 11. Toxicity

The over-supplementation or overconsumption of vitamin C has also been observed in some cases. The literature reports several side effects of ascorbic acid. Ingesting large doses of vitamin C (in gram quantities) can cause false-negative results in stool guaiac tests [44], as well as diarrhea and abdominal bloating. Studies have also found a correlation between vitamin C intake (from diet and supplements) and oxalate kidney stones in males, particularly at high doses [45]. Therefore, routine supplementation with vitamin C is not recommended for males, especially those who are predisposed to form oxalate stones. Such individuals should limit their intake of vitamin C to the recommended dietary allowance (RDA) in the United States.

There have been rare reports of fatal cardiac arrhythmias in patients with iron overload who ingested large amounts of ascorbic acid. This is thought to be due to oxidative injury [46]. Therefore, it may be advisable for patients to avoid taking pharmacologic doses of ascorbic acid supplements. However, there is no reason to discourage the consumption of fresh fruits or vegetables that contain vitamin C.

The LOVIT (Lessening organ dysfunction with Vitamin C) trial concluded that septic ICU patients who received a 4-day course of intravenous vitamin C had a higher risk of death or persistent organ dysfunction compared to those who received a placebo [47]. An interesting paper analyzed the data from the LOVIT trial to attempt to determine the cause of the higher deaths and organ dysfunction in the vitamin C group. They concluded that the increased mortality may be due to the abrupt termination of the ascorbic acid supplementation rather than the administration itself [48]. It is important for clinicians to thus be cognizant that the sudden halt of ascorbic acid supplementation can mimic a severe deficiency and lead to worse outcomes.

## 12. Limitations

Although this article has attempted to provide a comprehensive and concise review of vitamin C and its deficiency, especially in relation to the gastrointestinal system, we acknowledged certain limitations of this review. The review does not delve into many details about the basic science and biochemistry of vitamin C since we preferred to focus on the clinical implications of the deficiency. Scurvy is primarily still a historical disease, with most of the literature pertaining to it being older, with limited newer literature. Hence, our article contains information from relatively older studies and a limited proportion of recent case reports on scurvy. The demographic data is most relevant to the United States and does not cover the nutritional status of vitamin C in developing countries in Asia and Africa where scurvy would be expected to be most prevalent.

## 13. Conclusions

In summary, it is crucial for healthcare professionals to recognize and understand the significance of scurvy as a nutritional deficiency that has been prevalent for centuries but is increasingly being diagnosed in modern times. This increase in incidence is mainly due to several factors such as poor dietary habits, alcoholism, low socio-economic status, obesity, and abdominal surgeries. Scurvy can affect various organ systems due to its involvement in several biochemical reactions that affect tissue structure. Therefore, it is important to be aware of its potential gastrointestinal manifestations, particularly gastrointestinal bleeding. In cases in which the cause of gastrointestinal bleeding is an uncontrolled mucosal ooze, a high index of suspicion is necessary. Empirical treatment with vitamin C is a viable option due to its low cost and safety profile, particularly in patients with a high suspicion of scurvy.

In conclusion, scurvy is a preventable disease that can have severe consequences if left untreated. As such, it is crucial to maintain a balanced and healthy diet that includes sufficient amounts of vitamin C. Health professionals should be vigilant about the signs and symptoms of scurvy, especially in at-risk patients, and should consider vitamin C supplementation in suspected cases to prevent further complications.

## Figures and Tables

**Table 1:** Summary of cases of gastrointestinal manifestations in scurvy.

	Study	Age (In Years)	Gender	Manifestations
1.	Ohta A. et al. [37]	40	Male	Hematochezia. Antral, duodenal, and rectal erythema and mucosal hemorrhage.
2.	Callus CA et al. [38]	61	Male	Upper gastrointestinal bleeding. Gingivitis and bruising.
3.	Antunes et al. [39]	40	Male	Bleeding gums and episodic hematochezia. Cecal and ascending colon intramucosal hemorrhages.
4.	Ertugrul et al. [40]	56	Female	Refractory upper gastrointestinal bleeding, post-surgical state, mimicking portal gastropathy.
